# Effect of antiepileptic drug comedication on lamotrigine concentrations

**DOI:** 10.3325/cmj.2018.59.13

**Published:** 2018-02

**Authors:** Mila Lovrić, Ivana Čajić, Željka Petelin Gadže, Iva Klarica Domjanović, Nada Božina

**Affiliations:** 1 Department of Laboratory Diagnostics, University Hospital Center Zagreb, Zagreb, Croatia; 2 Department of Neurology, Referral Centre for Epilepsy, University Hospital Center Zagreb, Zagreb, Croatia; 3 School of Medicine, University of Zagreb, Zagreb, Croatia; 4 Agency for Medicinal Products and Medical Devices of Croatia, Zagreb, Croatia

## Abstract

**Aim:**

To estimate the effect size of concomitant antiepileptic therapy on the concentrations of lamotrigine, a drug often prescribed in combination with other antiepileptic drugs (AED), which can act as enzyme inducers or inhibitors.

**Methods:**

A total of 304 patients with epilepsy, aged 18-70 years, were divided into a lamotrigine monotherapy group and groups receiving lamotrigine with AEDs that act as enzyme inducers, enzyme inhibitors, or both. We compared lamotrigine monotherapy serum concentrations with those where lamotrigine was administered with a metabolic inhibitor valproate, metabolic inducers carbamazepine, oxcarbazepine, phenobarbital, phenytoin, or topiramate, and both an inducer and an inhibitor.

**Results:**

Comparison of trough lamotrigine monotherapy concentrations and lamotrigine polytherapy concentrations showed an almost similar median concentration in case of drug-inducers, and higher lamotrigine concentration in case of comedication with valproate as an inhibitor. A significant difference was confirmed after dose correction (*P* < 0.001). Significant positive correlations of lamotrigine trough serum concentrations with valproate were observed before and after the dose correction (r = 0.480, *P* < 0.001 and r = 0.561, *P* < 0.001, respectively). Positive correlations between the dose-corrected lamotrigine trough concentration and carbamazepine (r = 0.439; *P* < 0.001) or monohydroxy metabolite of oxcarbazepine (MHD) (r = 0.675; *P* < 0.001) were also significant.

**Conclusion:**

Higher valproate levels resulted in higher inhibition potency and higher lamotrigine levels. Increased dose-corrected concentrations of inducers carbamazepine and MHD, after the process of induction was finished, did not lower lamotrigine concentrations. These findings can be of clinical significance for optimal AED dosing.

Lamotrigine belongs to the second generation of antiepileptic drugs (AEDs) (1,2). Its metabolic interactions were recognized during development and before its marketing approval. As a result, a significant dose reduction was recommended and a wide therapeutic range was recognized in clinical practice (3). Lamotrigine is metabolized mainly by uridine-diphosphate-glucuronide transferase (UGT) 1A4, mostly to N2-glucuronide and to a minor N-5-glucuronide metabolite (4). Elimination of lamotrigine is age-related and highly influenced by liver or kidney function. Enzyme induction by other AEDs, such as carbamazepine, phenobarbital, phenytoin, and primidone, may result in increased lamotrigine clearance and decreased lamotrigine trough concentration, which requires dose corrections (5-7). Oxcarbazepine and topiramate may also induce lamotrigine metabolism. Valproate is a strong inhibitor of lamotrigine metabolism and may cause an increase in lamotrigine concentrations by 200% (5-9). According to Johannessen et al (10), lamotrigine and valproate pharmacokinetic interactions are classified as level 2 on the basis of magnitude of alterations in serum concentration, requiring caution and possible dose adjustments if the combination cannot be avoided. While lamotrigine metabolism occurs mainly via UGT1A4, glucuronidation of valproate is catalyzed primarily by UGT2B7 (11,12). UGTs are liable to both inhibition and induction, and the pharmacogenetic predisposition of UGTs has been broadly investigated in relation to their variability in drug metabolism (13,14).

Due to substantial pharmacokinetic variability of lamotrigine, there is a need for therapeutic drug monitoring. Furthermore, it has been observed that lamotrigine concentrations in the reference range, although rather wide, are not necessarily therapeutic, optimal, and effective. For some patients, individual therapeutic concentrations should be established. Our aim was to estimate the extent of the influence of other AEDs on lamotrigine concentrations in patients with focal and generalized seizures. This approach could help clinicians to estimate the possible correlation between drug interactions and disease control reflected in the reduced number of attacks and between drug interactions and side effects of antiepileptic drugs.

## METHODS

### Subjects

**Figure 1:**
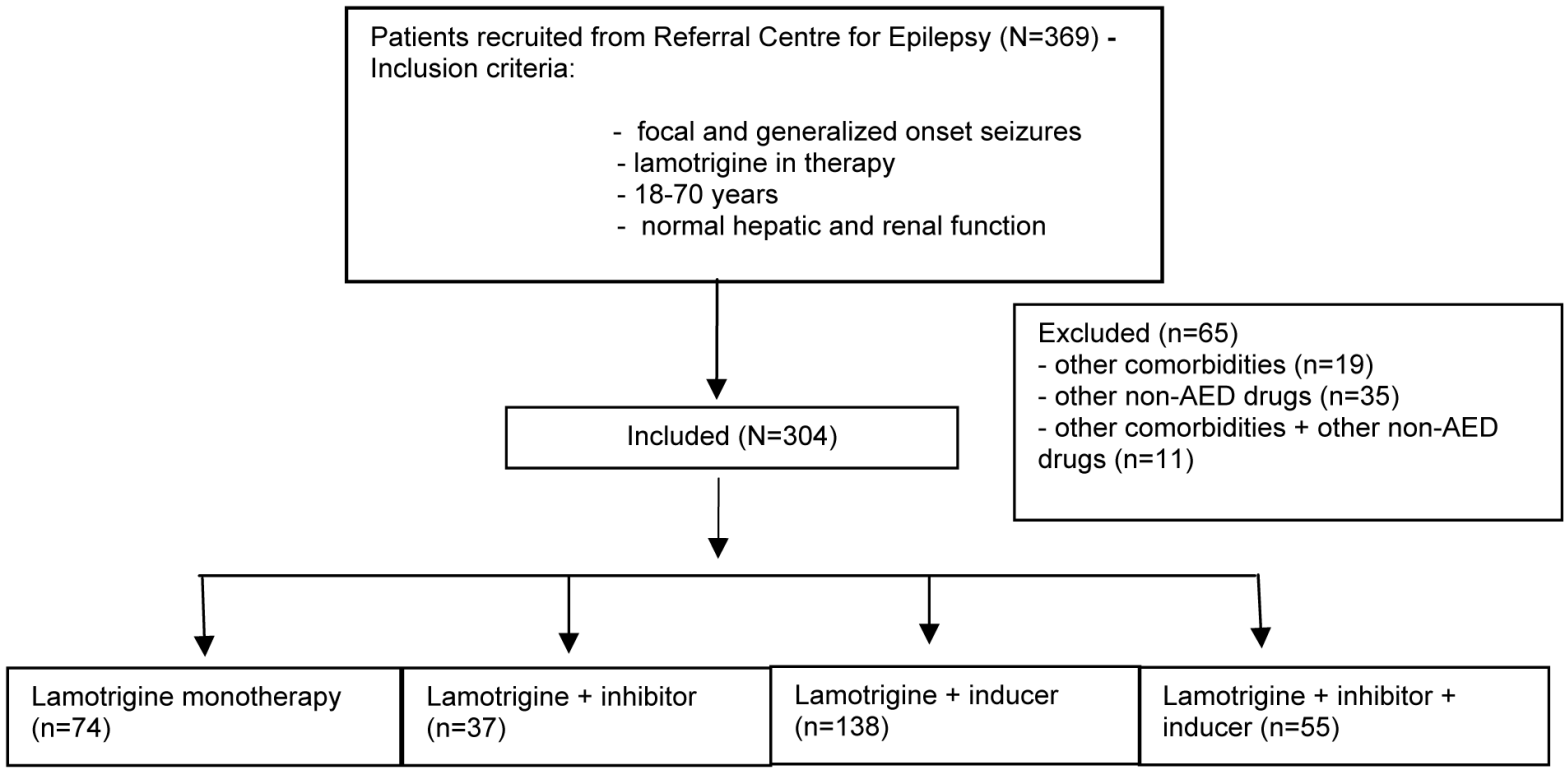
Flowchart of lamotrigine-treated patients with epilepsy included in the study.

This retrospective study included a cohort of 304 patients (126 men), aged 18-70 years, who were diagnosed as having epilepsy with focal or generalized seizures and treated at the Referral Centre for Epilepsy, University Hospital Center Zagreb (Figure 1). The exclusion criteria were impairment of hepatic and renal function as confirmed by standard biochemical parameters including urea, creatinine, and hepatic enzymes (Table 1). Data sources were patient medical histories from the University Hospital Center Zagreb, Department of Neurology and Department of Laboratory Diagnostics, from 2009 to 2013.

**Table 1:** Clinical and demographic data on 304 lamotrigine-treated patients (126 men) with epilepsy enrolled in the study*

Parameter	Value (mean±SD)
Age (years)	37 ± 14
Height (cm)	171 ± 9
Body weight (kg)	73 ± 18
Urea (mmol/L)	4.4 ± 1.4
Creatinine (μmol/L)	83.0 ± 17.4
ALT (U/L)	18.9 ± 15.1
GGT (U/L)	58.9 ± 49.9

*Abbreviations: SD – standard deviation, ALT – alanine aminotransferase, GGT – gamma-glutamyl transferase.

Lamotrigine was administered in doses from 50 to 300 mg/d as monotherapy in 74 patients or in combination with valproate (600 to 1500 mg/d) in 92 patients, carbamazepine (600-1600 mg/d) in 110 patients, oxcarbazepine (600-1800 mg/d) in 47 patients, phenobarbital (100-600 mg/d) in 78 patients, phenytoin (200-300 mg/d) in 9 patients or topiramate (50-250 mg/d) in 10 patients. Several patients were also treated with vigabatrin, gabapentin or levetiracetam. According to the known impact of each drug on the pharmacokinetics of lamotrigine, patients were divided into four groups as follows: patients receiving lamotrigine monotherapy, patients receiving lamotrigine in combination with a metabolic inhibitor (valproate), patients receiving lamotrigine in combination with metabolic inducers (carbamazepine, oxcarbazepine, phenobarbital, phenytoin, or topiramate), and patients receiving lamotrigine in combination with both an inducer and an inhibitor.

### Therapeutic drug monitoring

In the Referral Centre for Epilepsy, serum concentrations of antiepileptic drugs are routinely measured in each patient. This implies that all measurements were available for all patients included in the study.

One blood sample per patient was collected after a steady state concentration of the drug was achieved, ie, not less than 21 days from start of therapy. Blood samples were obtained in the morning, 12 hours after bedtime dose and before the morning dose (trough value). Serum concentrations of lamotrigine, carbamazepine, 10-monohydroxy metabolite of oxcarbazepine (MHD), and phenytoin were measured by high performance liquid chromatography methods with a diode array detector (Shimadzu, Kyoto, Japan). Sample and internal standard (chloramphenicol 100 µg/ml) were extracted with hexane-ethyl acetate (1:1). Drug compounds were separated on Nucleosil 100-5 C18 column (Macherey Nagel, Düren, Germany) with the mobile phase acetonitrile/methanol/0.05 M NaH_2_PO_4_*H^2^O, pH 6.4 buffer (6:5:19, v/v/v). The eluted substance was detected at 306 nm, 234 nm, and 204 nm (15). Serum topiramate concentrations were analyzed using fluorescence polarization immunoassay Innofluor (Seradyn Inc., Indianapolis, IN, USA) on a TDx (Abbott Laboratories, Lake Bluff, IL, USA) analyzer. Vigabatrin and gabapentin were measured after derivatization by high performance liquid chromatography (HPLC) method using a fluorescence detector (Waters, Milford, MA, USA). The serum concentrations of phenobarbital and valproate were analyzed by immunoassay on a Dimension Expand analyzer (Siemens, Erlangen, Germany). The calibrator and control samples used for HPLC method were from Chromsystems (Munich, Germany), whereas those used for the immunoassay were from Innofluor and Siemens. All analytes were evaluated through the external quality assessment schemes (Referenzinstitut für Bioanalytik and United Kingdom National External Quality Assessment Service).

### Statistical analysis

Descriptive statistics was used for demographic and clinical data presentation. After testing for normality of data distribution with D’Agostino-Pearson test, differences in steady state trough and dose-corrected lamotrigine concentrations between therapy groups were analyzed. As the lamotrigine concentration was not normally distributed in the study population, significance of differences between the groups was tested with the Kruskal-Wallis test. Correlation analysis between parameters was performed by Spearman’s coefficient of rank correlation and presented as correlation coefficients (rho values) with 95% confidence interval (CI) and *P* values. The level of statistical significance was set at *P* < 0.05. All statistical analyses were performed using the statistical program MedCalc ver. 17.2.

## RESULTS

**Figure 2:**
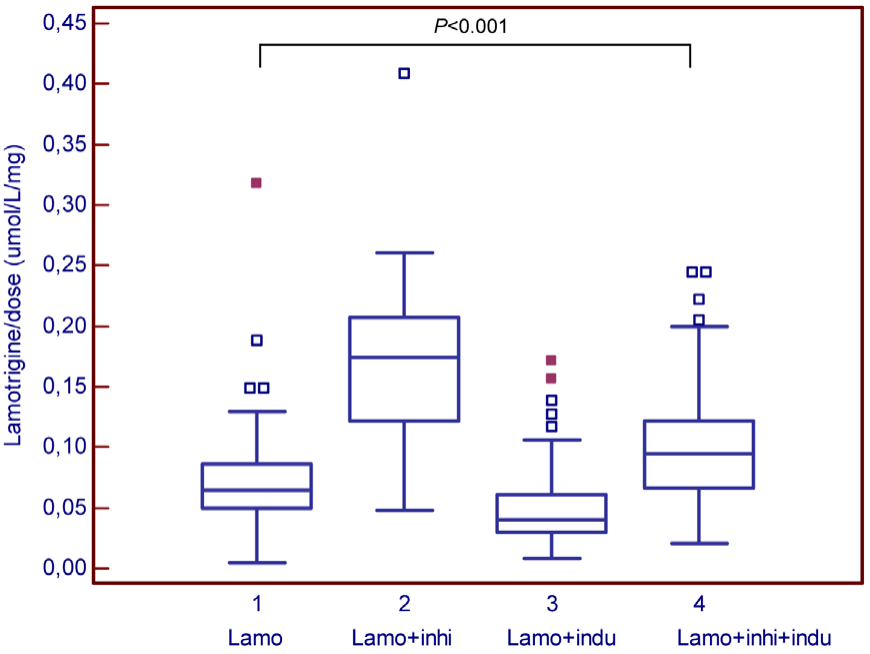
Dose-corrected concentrations of lamotrigine administered with concomitant antiepileptic drugs acting as inhibitors or inducers. Abbreviations: Lamo – lamotrigine, inhi – inhibitor, indu – inducer.

All median values of lamotrigine trough concentrations lay within the reference range (8), but median concentrations of majority of other AEDs were closer to the lower range area (Table 2). There were significant differences in trough lamotrigine and dose-corrected lamotrigine concentrations between the four groups (*P* < 0.001; Figure 2.). Patients receiving lamotrigine with valproate had the highest concentrations of lamotrigine even after the dose correction of the concentration (Table 2). Patients on lamotrigine monotherapy had the lowest lamotrigine concentration. However, after the dose correction, the lowest values were observed in patients treated with lamotrigine in combination with one of the metabolic inducers.

**Table 2:** Serum trough- (C) and dose-corrected (C/D) antiepileptic drug (AED) concentrations in lamotrigine-treated patients with epilepsy*

			AED concentrations (median, 5th-95th percentile)	
Therapy	No. of patients	f	C (µmol/L)	C/D (µmol/L/mg)
Lamotrigine	304		8.6 (2.3-38.9)	0.062 (0.022-0.202)
monotherapy	74		6.4 (1.6-23.5)	0.065 (0.024-0.145)
+ inhibitor	37	3.90	25.9 (5.3-50.8)	0.174 (0.059-0.261)
+ inducer	138		6.5 (2.0-21.7)	0.040 (0.017-0.098)
+ inhibitor and inducer	55		15.8 (3.0-57.3)	0.094 (0.044-0.218)
politherapy				
+ valproate	92	6.93	339.7 (128.9-652.9)	0.342 (0.154-0.603)
+ phenobarbital	78	4.31	69.4 (14.1-168.6)	0.246 (0.033-0.673)
+ carbamazepine	110	4.23	32.7 (18.4-53.1)	0.032 (0.018-0.073)
+ MHD	47	3.96	58.0 (22.6-97.7)	0.046 (0.018-0.111)

*Abbreviations: f – conversion factor (µmol/L = mg/L x f) (8), MHD – monohydroxy metabolite of oxcarbazepine.

Lamotrigine concentrations varied depending on the dose and concomitant therapy. We evaluated the correlation between lamotrigine and valproate in all patient groups concomitantly treated with these two drugs and the correlation of lamotrigine with carbamazepine, MHD, and phenobarbital in patients without concomitant valproate therapy.

There was a significant linear positive correlation between lamotrigine and valproate trough concentrations, which was even greater after the dose correction (Table 3). Comparison of lamotrigine with MHD and phenobarbital did not show any correlation for trough concentrations, but a significantly positive correlation was observed for carbamazepine and MHD after the dose correction. Nine patients in the groups receiving lamotrigine in combination with inducers or both inhibitors and inducers had phenytoin in their therapy, but statistical analysis was not performed due to the low number of these patients.

**Table 3:** Correlation of serum trough- and dose-corrected concentrations of lamotrigine administered with concomitant antiepileptic drug (AED) therapy

		Lamotrigine (μmol/L)		Lamotrigine/dose (μmol/L/mg)	
AED	No. of patients	rho (95% CI)*	*P*	rho (95% CI)*	*P*
Valproate	87	0.480 (0.298-0.628)	<0.001	0.561 (0.383-0.699)	<0.001
Carbamazepine	85	0.367 (0.166-0.538)	<0.001	0.439 (0.235-0.594)	<0.001
Phenobarbital	56	0.229 (0.036-0.464)	0.09	0.051 (-0.247-0.304)	0.83
MHD	33	0.228 (-0.125-0.530)	0.20	0.675 (0.415-0.833)	<0.001

*rho –Spearman’s coefficient of rank correlation with 95% confidence intervals (CI).

## DISCUSSION

Our results confirmed the inhibitory effect of valproate with the resulting increase in lamotrigine concentrations. Despite the dose reduction at the introduction of therapy, the observed lamotrigine blood concentrations were still higher than those when lamotrigine was administered as monotherapy or concomitantly with other AEDs. This finding has a clinical importance due to dose-dependent side effects. We found a statistically significant correlation between lamotrigine and valproate trough concentrations. The correlation was statistically even stronger for dose-corrected concentrations, which is not fully consistent with literature data. As valproate can double lamotrigine plasma levels, some authors recommend that the initial dose should be reduced approximately by 50%. Kanner et al (16) reported that the degree of inhibition of lamotrigine clearance is independent of the applied valproate dose and steady state valproate concentration. Some authors suggest that valproate inhibition of lamotrigine clearance can be expected at a dose of 500 mg per day, with the magnitude of inhibition diminishing at lower doses (17). However, these results were obtained in healthy volunteers who received rather low doses of valproate. Yamamoto et al (18) showed that valproate strongly inhibits lamotrigine metabolism, but they did not observe a significant correlation between plasma lamotrigine concentrations adjusted to dose and valproate concentrations. These discordant results reported for different populations and ethnic groups may be partly explained by significant differences in variant allele frequencies of the *UGT2B7* gene, ie, the frequency of the UGT 2B7*2 (802C>T) variant has been reported to be 48.9%-53.7% in Caucasians and 24.4%-29.3% in Japanese population (19).

When we tested the correlations of lamotrigine with a single antiepileptic drug inducer such as carbamazepine, MHD, and phenobarbital, the results indicated that the trough lamotrigine concentrations were not in correlation with trough concentrations of MHD or phenobarbital. Because patients were treated with different doses of AEDs, a dose correction of the concentration was undertaken, after which the correlation reached statistical significance for carbamazepine and MHD. Oxcarbazepine may stimulate a more restricted range of CYP and/or UGT isoenzymes with weaker enzyme-inducing properties (20). Positive correlation between lamotrigine and MHD, the main metabolite of oxcarbazepine, may be explained by the main mechanism of MHD elimination, which is primarily by glucuronide conjugation. The primary metabolic route for carbamazepine is oxidation, which produces an epoxide that is subsequently further oxidized to a diol (21). This is followed by conjugation with UGT2B7, which can explain the weak correlation between lamotrigine and carbamazepine. The induction of lamotrigine metabolism by carbamazepine or oxcarbazepine may result in decreased lamotrigine concentrations, but in the next step, carbamazepine/MHD and lamotrigine compete for the same enzymes. This can finally result in increased concentrations of both lamotrigine and carbamazepine/MHD (22).

We observed no effect on lamotrigine kinetics, as previously described (23), of topiramate, which is a weak inducer (24), and others such as gabapentin, vigabatrin, and levetiracetam whose main route of elimination is renal excretion. This could be due to the small number of patients in each group, which did not allow drawing any firm conclusions.

Due to complexity of these mechanisms, it is still difficult to predict the final outcome of these interactions (25). Furthermore, drug transporters, present at many barriers and organs involved in drug absorption, distribution, and excretion, play a key role in the bioavailability and concentrations of many drugs, including AEDs. Additionally, the fact that drugs can be substrates and inhibitors or inducers of transporter proteins makes the pharmacokinetics of AEDs even more complex (26).

In conclusion, our original finding was that higher valproate concentration levels resulted in higher lamotrigine serum levels. This is a fact that clinicians should keep in mind when concomitantly prescribing these two drugs, since majority of their adverse effects are dose-dependent. Additionally, significant positive correlations between lamotrigine, carbamazepine, and MHD concentrations indicated that upon the completion of induction, a higher dose-corrected concentration of inducers did not further lower lamotrigine levels. These findings may have clinical significance for optimal AED dosing, since side effects of AEDs are dose-dependent and reinforce the view that optimizing lamotrigine dose in an individual patient is best achieved by adjunctive measurement of serum levels. More studies with larger sample sizes than those in our study are needed to validate our findings.
